# Socioeconomic and geographic inequalities in colorectal cancer in Cyprus: An ecological study

**DOI:** 10.1093/eurpub/ckac131.102

**Published:** 2022-10-25

**Authors:** D Lamnisos, K Giannakou

**Affiliations:** Department of Health Sciences, European University Cyprus, Nicosia, Cyprus

## Abstract

**Background:**

Colorectal cancer (CRC) is one of the main causes of mortality and morbidity worldwide. To date, the relationship between regional deprivation and CRC incidence or mortality has not been studied in the population of Cyprus. The aim of this study was to analyse the geographical variation of CRC incidence and mortality and its possible association with socio-economic inequalities in Cyprus for the periods between 2000 and 2015.

**Methods:**

A small area ecological study in Cyprus, with census tracts as units of spatial analysis, for the period between 2000 and 2015. The incidence date, sex, age, post code, primary site, death date in case of death or last contact date in case of alive for all cases of CRC from 2000-2015 were obtained from the Cyprus Ministry of Health. Indirect standardization was used to calculate the sex and age Standardize Incidence Ratios (SIRs) and Standardized Mortality Ratios (SMRs) of CRC while the smoothed values of SIRs, SMRs and Mortality to Incidence ratio (M/I ratio) were estimated using the univariate Bayesian Poisson log-linear spatial model.

**Results:**

There are geographical areas having 15% higher SIR and SMR, with most of those areas located at the east coast of the island. Higher M/I ratio values were found in the rural, remote, and less dense areas of the island while lower rates were observed in the metropolitan areas. An inverted U-shape pattern in CRC incidence and mortality was observed with higher rates in the areas classified in the second quartile of the socio-economic deprivation index and lower rates in rural, remote, and less dense areas. A different pattern emerged in the M/I ratio indicating a stepwise increase across increasing levels of socioeconomic deprivation.

**Conclusions:**

These findings can potentially provide useful information at local and national levels and can inform public health authorities to appropriately allocate resources for geographically targeted prevention and control plans to increase CRC screening.

**Key messages:**

• M/I ratio of CRC was positively associated with regional deprivation since a stepwise increase was found across increasing levels of rural-related socioeconomic deprivation.

• Interventions aimed at reducing the risks of CRC should primarily focus on socially deprived communities in Cyprus.

